# Cardiovascular Risk Assessment in a Cohort of Newly Diagnosed Patients with Obstructive Sleep Apnea Syndrome

**DOI:** 10.1155/2018/6572785

**Published:** 2018-03-08

**Authors:** Kostas Archontogeorgis, Athanasios Voulgaris, Evangelia Nena, Maria Strempela, Panagiota Karailidou, Argyrios Tzouvelekis, Toulin Mouemin, Maria Xanthoudaki, Stylianos Steiropoulos, Marios E. Froudarakis, Paschalis Steiropoulos

**Affiliations:** ^1^MSc Program in Sleep Medicine, Medical School, Democritus University of Thrace, Alexandroupolis, Greece; ^2^Department of Pneumonology, Medical School, Democritus University of Thrace, Alexandroupolis, Greece; ^3^Laboratory of Hygiene and Environmental Protection, Medical School, Democritus University of Thrace, Alexandroupolis, Greece; ^4^Division of Immunology, Biomedical Sciences Research Center “Alexander Fleming”, Athens, Greece; ^5^1st Cardiology Department, General Hospital of Nikea “Agios Panteleimon”, Nikea, Greece

## Abstract

**Objectives:**

Obstructive sleep apnea syndrome (OSAS) is associated with increased cardiovascular morbidity and mortality. The aim of this study was to assess whether the 10-year risk for cardiovascular disease in newly diagnosed patients with OSAS is increased.

**Materials and Methods:**

Recently diagnosed, with polysomnography, consecutive OSAS patients were included. The Systematic Coronary Risk Evaluation (SCORE) and the Framingham Risk Score (FRS) were used to estimate the 10-year risk for cardiovascular disease.

**Results:**

Totally, 393 individuals (73.3% males), scheduled to undergo a polysomnographic study with symptoms indicative of OSAS, were enrolled. According to apnea-hypopnea index (AHI), subjects were divided in four groups: mild OSAS (AHI 5–14.9/h) was diagnosed in 91 patients (23.2%), moderate OSAS (AHI 15–29.9/h) in 58 patients (14.8%), severe OSAS (AHI > 30/h) in 167 patients (42.5%), while 77 individuals (19.6%) had an AHI < 5/h and served as controls. Increased severity of OSAS was associated with increased SCORE (*p* < 0.001) and FRS values (*p* < 0.001). More specifically, a significant correlation was observed both between AHI and SCORE (*r*=0.251,  *p* < 0.001) and AHI and FRS values (*r*=0.291,  *p* < 0.001). Furthermore, a negative correlation was observed between FRS values and sleep efficiency (*r*=−0.224,  *p*=0.006).

**Conclusions:**

The 10-year risk for cardiovascular morbidity and mortality seems to increase with severity of OSAS. Physicians should bear this finding in mind, in order to seek for and consecutively eliminate risk factors for cardiovascular disease and to prevent future cardiovascular events in OSAS patients.

## 1. Introduction

Obstructive sleep apnea syndrome (OSAS) is characterized by recurrent episodes of partial or complete collapse of the upper airway during sleep, leading to oxyhaemoglobin desaturation, frequent arousals, and sleep fragmentation [1]. It is a common disorder, with prevalence estimated at 10–17% for men and 3–9% for women, among adults in the developed countries [2]. OSAS is associated with excessive daytime sleepiness, poor quality of life, reduced work productivity [3], increased overall morbidity, and mortality [4]. Evidence also supports the relationship between OSAS and arterial hypertension, coronary heart disease, heart failure, and arrhythmias [5, 6]. Previous reports suggest that heart disorders occur in approximately 40% of patients with OSAS [5]. Intermittent hypoxia triggered by apneic events, as well as abrupt reduction of the intrathoracic pressure and augmentation of the sympathetic tone, contribute to the pathogenesis of cardiovascular disease in OSAS [7]. However, OSAS and cardiovascular disease share common risk factors, such as obesity, hypertension, and diabetes [8]. Thus, the association between OSAS and cardiovascular disease may be attributed to either a clustering of risk factors present in both conditions, or to a real causal role. The aim of this study was to evaluate whether the estimated 10-year risk for cardiovascular morbidity and mortality, as assessed by Framingham Risk Score (FRS) and Systematic Coronary Risk Evaluation (SCORE), respectively, is increased in recently diagnosed patients with OSAS and to explore possible associations between those cardiovascular assessment risk scores with polysomnographic characteristics in OSAS.

## 2. Patients and Methods

Included were consecutive individuals referred to the sleep laboratory of our institution in a 6-month period, between January and June 2015, with symptoms suggestive of sleep-related breathing disorders. The study was performed in accordance with the Helsinki Declaration of Human Rights, as revised in 2013 [9], and patients provided their informed consent.

Exclusion criteria were age > 65 years, history of stroke, cardiovascular disease and arrhythmias, chronic kidney disease, use of statins or aspirin, any chronic inflammatory disease, exclusively central apneas on polysomnography, diabetes, and endocrine disorders.

Information on previous medical history, current medication use, tobacco smoking, and alcohol consumption were recorded. Height, weight, body mass index (BMI) (weight (kg)/height (m)^2^), as well as neck, waist, and hip circumference and waist/hip ratio were measured using a standardized protocol.

Blood pressure was recorded in the sitting position after a 10 minute rest, as the average of 3 consecutive measurements separated by a 1 minute interval. Diagnosis of diabetes mellitus was based either on known medical history of diabetes or measurement of fasting plasma glucose ≥126 mg/dl [10].

Sleepiness was evaluated by the validated version of the Epworth sleepiness scale (ESS) in Greek language [11], a self-administered questionnaire evaluating the possibility of falling asleep in a variety of situations (maximum score: 24; score >10 indicative of excessive daytime sleepiness). Pulmonary function testing, arterial blood gasses, and a 12-lead electrocardiographic examination before sleep study were also performed.

### 2.1. Polysomnography

Overnight polysomnography (PSG) (Alice® 4, Philips Respironics, Murrysville, PA, USA), attended by an experienced technician, was performed from 22 : 00 to 06 : 00 hours. A standard montage of electroencephalogram, electroocculogram, electromyogram, and electrocardiogram signals was used. Pulse oximetry was registered and airflow was detected using combined oronasal thermistors. Thoracic cage and abdominal motion were recorded using inductive plethysmography, as previously described [12]. The polysomnographic recordings were manually scored according to the 2007 American Association of Sleep Medicine guidelines for the scoring of sleep and associated events [13]. The proportion of time spent in each stage of sleep was calculated as a percentage of total sleep time. Apnea was defined as 90% reduction in airflow for at least 10 sec [13]. Hypopnea was defined as a 30% reduction in airflow for at least 10 sec in combination with oxyhaemoglobin desaturation of at least 3% or an arousal registered by the electroencephalogram [13]. A central apnea was scored in the presence of cessation of airflow for 10 seconds or longer without an identifiable respiratory effort [13]. The apnea-hypopnea index (AHI) was calculated as the average number of apneas and hypopneas per hour of PSG-recorded sleep time [13]. Oxygen desaturation index (ODI) was calculated as the average number of arterial oxygen desaturations ≥ 3% per hour of sleep [13]. Sleep efficiency was defined as the ratio of total sleep time (TST) to time in bed (multiplied by 100 to yield a percentage).

OSAS was diagnosed when AHI was >5/h, accompanied by related symptoms, and was graded as mild (AHI: 5–14.9/h), moderate (AHI: 15–29.9/h), or severe (AHI: >30/h) [1].

### 2.2. Blood Samples

All blood samples were collected after at least 8 hours of overnight fasting, were immediately centrifuged (10 minutes at 3000 rpm), and were preserved at −80 C° until analysis. Fasting blood glucose, triglycerides, total cholesterol, and high- and low-density lipoprotein were calculated by a random-access chemistry analyzer (AU640; Olympus; Hamburg, Germany).

### 2.3. Cardiovascular Risk Assessment

The 10-year risk for cardiovascular disease was assessed using two validated prediction models: the low-risk Systematic Coronary Risk Evaluation (SCORE) and the Framingham Risk Score (FRS). The SCORE estimates the 10-year risk of fatal cardiovascular disease using an algorithm that considers parameters such as age, gender, total cholesterol, high-density lipoprotein cholesterol, systolic blood pressure, and smoking status [14]. Combining those parameters, SCORE values are reflecting the 10-year risk for cardiovascular mortality from a range of lower than 1% to greater than 15%. The FRS, which assesses the 10-year risk of developing cardiovascular disease in general and in specific of its components such as coronary heart disease, cerebrovascular disease, and heart failure, was calculated considering risk factors such as age, gender, total cholesterol and high-density lipoprotein cholesterol, systolic blood pressure, hypertension treatment, smoking status, and diabetes [15]. Risk groups were defined by risk percentages as follows: low  <10%, intermediate 10–20%, high >20%, and obtained only in patients without a history of cardiovascular disease.

### 2.4. Statistical Analysis

Analysis was performed using Statistical Package for Social Sciences version 17.0 (SPSS Inc. Released 2008. SPSS Statistics for Windows, Version 17.0. Chicago: SPSS Inc.). Continuous variables were tested for normality of distribution by the Kolmogorov–Smirnov test. Quantitative data with normal distribution were expressed as mean ± standard deviation (SD) and with skewed distribution as median (25th–75th percentile). Correlations were analyzed with Pearson's correlation coefficient, while in case of skewed distribution, the Spearman correlation coefficient was applied. Comparisons between means were explored with the Student's *t*-test, and for skewed distribution, the nonparametric Mann–Whitney test was applied. Associations between variables on univariate regression analysis, with a two-tailed *p* < 0.05, were entered into multivariate models to determine the independent correlations with cardiovascular risk scores.

## 3. Results

A total of 393 patients (288 males and 105 females) were included in the study. Patients were stratified, according to AHI, in 4 groups: control group (AHI < 5 events/h), including 77 subjects (41 males and 36 females); mild OSAS (AHI 5–14.9 events/h), including 91 patients (64 males and 27 females); moderate OSAS (AHI 15–29.9 events/h), including 58 patients (42 males and 12 females); and severe OSAS (AHI > 30 events/h), including 167 patients (141 males and 26 females). Mean age of the control group was significantly lower than that of any subgroup of OSAS patients (45.3 ± 10.1 for controls versus 48.8 ± 9.8 for mild OSAS, *p*=0.023; versus 53 ± 9.8 for moderate OSAS, *p* < 0.001; and versus 51 ± 9.7 years for severe OSAS, *p* < 0.001) while patients with severe OSAS had higher BMI compared with controls (35.5 ± 6.6 versus 32.5 ± 7.4 kg/m^2^, *p*=0.002). Anthropometric characteristics of participants are presented in Table 1, while sleep characteristics are presented in Table 2.

In all OSAS groups, mean systolic blood pressure was higher compared to controls (Table 3). In severe OSAS, increased serum cholesterol and high-density lipoprotein cholesterol levels compared with controls were observed (Table 3).

**Figure 1:**
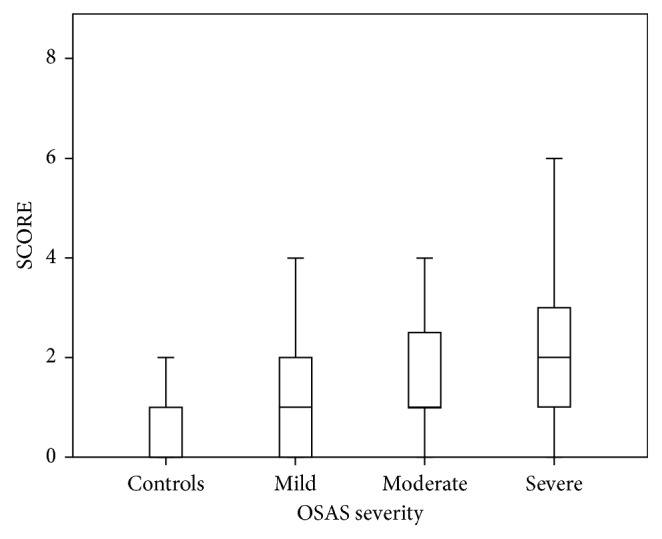
Comparison of Systematic Coronary Risk Evaluation (SCORE) score between controls and obstructive sleep apnea syndrome (OSAS) patients.

SCORE increased in line with OSAS severity and was higher compared to controls and among OSAS groups of increasing severity (0 (0-1) for controls versus 1 (0–2) for mild OSAS, *p*=0.001; versus 1 (0–3) for moderate OSAS, *p* < 0.001; and versus 1.5 (1–3) for severe OSAS, *p* < 0.001) (Figure 1).

**Figure 2:**
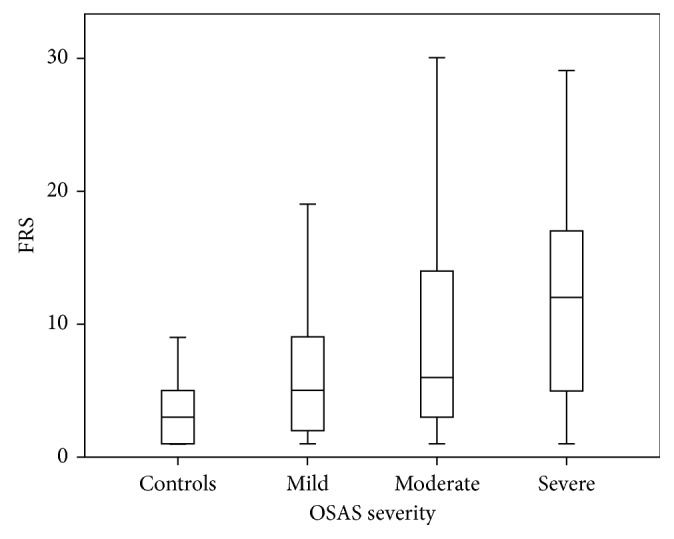
Comparison of Framingham Risk Score (FRS) between controls and obstructive sleep apnea syndrome (OSAS) patients.

Likewise, FRS increased in parallel with OSAS severity and was higher in OSAS patients than that of the control group (3 (1–5) for the control group versus 4 (1.5–7) for mild OSAS, *p*=0.004; versus 10 (2.5–13.5) for moderate OSAS, *p* < 0.001; and versus 12 (5–15) for severe OSAS, *p* < 0.001) (Figure 2). Results of laboratory analyses and measurements performed are presented in Table 3.

**Figure 3:**
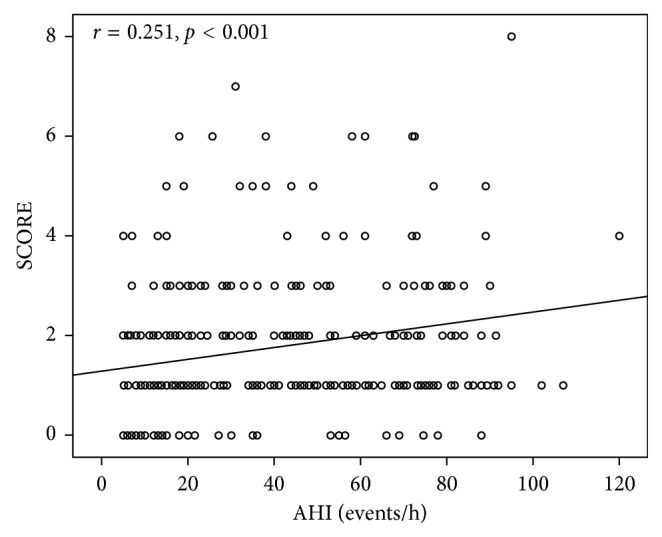
Correlation between Systematic Coronary Risk Evaluation score (SCORE) and apnea-hypopnea index (AHI) in obstructive sleep apnea syndrome patients.

**Figure 4:**
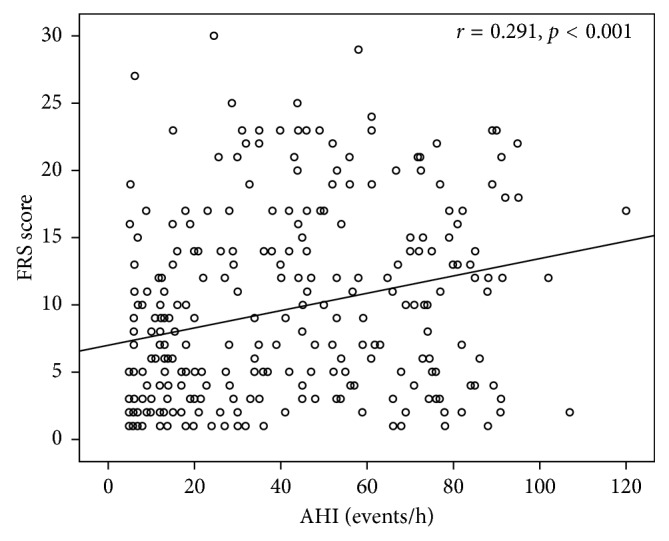
Correlation between Framingham Risk Score (FRS) and apnea-hypopnea index (AHI) in obstructive sleep apnea syndrome patients.

**Figure 5:**
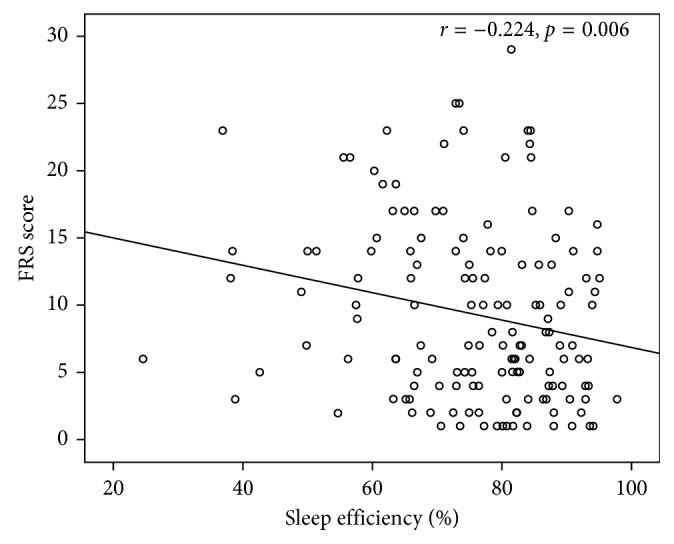
Association between Framingham Risk Score (FRS) and sleep efficiency in obstructive sleep apnea syndrome patients.

In OSAS patients, a positive correlation between AHI and SCORE was revealed (*r*=0.251,  *p* < 0.001) (Figure 3) as well as between AHI and FRS (*r*=0.291,  *p* < 0.001) (Figure 4) and a negative correlation between FRS and sleep efficiency (*r*=−0.224,  *p*=0.006) (Figure 5). Results of the conducted correlation analyses between SCORE, FRS, anthropometric, and sleep parameters are presented in Table 4.

After adjustment for age and BMI, SCORE was significantly correlated with age (*β*=0.587,  *p* < 0.001), BMI (*β*=0.131,  *p*=0.005), and AHI (*β*=0.257,  *p* < 0.001), while FRS was correlated with age (*β*=0.219,  *p* < 0.001), BMI (*β*=−0.228,  *p* < 0.001), and AHI (*β*=0.334,  *p* < 0.001).

## 4. Discussion

The current study demonstrates that the 10-year risk for cardiovascular morbidity and mortality, as expressed by the two multivariate risk models, FRS and SCORE, respectively, increases in accordance with OSAS severity. Moreover, an inverse association between the 10-year risk for cardiovascular disease and sleep efficiency was noticed.

FRS and SCORE constitute multivariate risk models which are assessing the 10-year risk for cardiovascular disease in individuals presented without established cardiovascular disease. Both point out the need of primary prevention for patients who are at high risk for future cardiovascular events, either fatal or nonfatal, such as coronary heart disease, stroke, heart failure, and intermittent claudication [14, 15]. Patients, who found to have an increased likelihood of cardiovascular morbidity and mortality, as assessed by those risk models, should receive hypertensive treatment, start on statin and aspirin therapy, and apply lifestyle modifications.

OSAS has been previously associated with increased morbidity and mortality related to cardiovascular disease, particularly coronary heart disease and stroke [5, 7, 8]. In a study [16] that included 114 OSAS patients (100 males and 14 females), 10-year risk for coronary heart disease in males was 13.9 ± 0.9%, (95% CI: 12.1–16.0) and for stroke was 12.3 ± 1.4%; (95% CI: 9.4–15.1), with a combined 10 year risk for stroke and CHD events of 32.9 ± 2.7%; (95% CI: 27.8–38.5) in males aged > 53 years. In the same study, when patients were stratified according to their AHI, coronary heart disease risk did not significantly differ among groups [16]. However, in this study, HDL-C levels were not measured and were not considered in the risk prediction model; thus, results may tend to underestimate the true cardiovascular risk related to abnormal lipid status.

Similarly to our results, in a population-based study that included 904 patients classified according to FRS score, AHI was increased in the high-risk group compared to the intermediate- and low-risk groups (23.9 ± 2.8 versus 17.7 ± 1.8 versus 7.2 ± 0.5 events/h, resp., *p* < 0.001) [17]. Sleep efficiency also differed between groups (67.6 ± 2.5% for the high-risk group; 78.4 ± 1.6% for the intermediate-risk group; and 82.9 ± 0.4% for the low-risk group, resp., *p* < 0.001) [17]. After adjustment for confounding factors, age (*p* < 0.001) and sleep efficiency (*p*=0.006) remained strongly associated with high-risk patients [17].

Wang et al. [18] divided 120 OSAS patients in 2 groups according to FRS score: low-risk group (with FRS < 10) and non-low-risk group (with FRS ≥ 10). Subjects with higher FRS had a larger neck circumference (40.48 ± 2.80 versus 39.15 ± 4.31 cm, *p* < 0.05), longer course of disease (12.77 ± 7.89 versus 9.36 ± 5.98 years, *p* < 0.05), increased AHI (47.61 ± 25.63 versus 34.01 ± 25.72 events/h, *p* < 0.01), lower minimum oxyhaemoglobin saturation during sleep (73.85 ± 11.10 versus 77.91 ± 9.77%, *p* < 0.05), longer time with oxyhaemoglobin saturation <90% (23.46 ± 24.46 versus 14.48 ± 18.85%, *p* < 0.05), and higher ODI (49.62 ± 23.75 versus 39.01 ± 24.87 events/h, *p* < 0.05) [18]. AHI was the most important determining factor for FRS (*t*=2.910,  *p*=0.004) [18].

In our study, an association between FRS score and sleep efficiency, an objective index of sleep quality, was observed. A number of studies examined the association between sleep efficiency and arterial blood pressure, often reporting conflicting results [19, 20]. Good sleep efficiency, defined as sleep efficiency >90%, was less frequently observed among OSAS patients with cardiovascular disease (OR 0.45 (0.22–0.91)) [21].

In a study [22] that compared systolic arterial pressure and heart rate, nocturnal dipping between subjects with low and high sleep efficiency demonstrated that the group with low sleep efficiency presented a blunted dip of nocturnal systolic arterial pressure (10 ± 1 versus 14 ± 1%, *p*=0.04) and heart rate (12 ± 3 versus 21 ± 3%, *p*=0.03) compared with the high sleep efficiency group. Additionally, the low sleep efficiency group had higher mean nocturnal heart rate (63 ± 2 versus 55 ± 2 beats/min, *p*=0.02) [21]. In women, poor sleep quality has been associated with resistance to hypertensive treatment [23]. In the present study, sleep efficiency markedly decreased in the severe OSAS group, and in the same group, the percentage of slow-wave sleep was also reduced. Previous data showed that patients with reduced slow-wave sleep had decreased sympathetic activity during slow-wave sleep and increased risk of developing hypertension among elderly men [24, 25]. Thus, our findings further support the notion that sleep efficiency may be an important factor to be considered regarding the role of inadequate sleep in cardiovascular disease.

Certainly, our study is subject to some limitations. First, there was an over-representation of patients with severe OSAS in our sample, which was attributed to the distribution of subjects referred in a sleep laboratory with complaints of sleep-disordered breathing and increased likelihood of sleep apnea. We also acknowledge the fact that the performance of a single night PSG may influence our results since a single night recording has been associated with reduced sleep efficiency and fragmented sleep [26]. Moreover, evaluation of cardiovascular risk using these models is limited in a period time of only 10 years while the mean age of our sample size was relatively small. Thus, long-term cardiovascular assessment of young OSAS patients with lifetime risk models should be part of routine evaluation and preferred instead of the 10-year risk model. Finally, we did not examine the potential beneficial effect of continuous positive airway pressure (CPAP) treatment on those risk models, which could be possibly modified, especially in patients with good adherence, as it was demonstrated with single biomarkers associated with increased cardiovascular risk [27]. However and despite the above mentioned limitations, one of the major strengths of the present study is that it has used two different tools to evaluate cardiovascular risk, which resulted in similar findings, verifying the applicability of the selected models in our study [28].

In conclusion, the 10-year risk for cardiovascular morbidity, in a cohort of newly diagnosed OSAS patients, increases along with the severity of OSAS as indicated by AHI. Sleep physicians should bear this finding in mind, in order to seek for and eliminate risk factors for cardiovascular disease and to prevent future cardiovascular events in patients with OSAS. Further investigation on cardiovascular risk assessment based upon risk models with large epidemiological studies in OSAS patients is needed.

## Figures and Tables

**Table 1:** Comparison of anthropometric characteristics between controls and obstructive sleep apnea syndrome (OSAS) patients.

	Controls	Mild OSAS	Moderate OSAS	Severe OSAS	Controls versus mild OSAS	Controls versus moderate OSAS	Controls versus severe OSAS	Mild versus moderate OSAS	Mild versus severe OSAS	Moderate versus severe OSAS
Number	77	91	58	167	—	—	—	—	—	—
Gender (males/females)	41/36	64/27	42/16	141/26	**0.023**	**0.020**	**<0.001**	0.736	**0.007**	**0.05**
Age (years)	45.3 ± 10.1	48.8 ± 9.8	53 ± 9.8	51 ± 9.7	**0.023**	**<0.001**	**<0.001**	**0.011**	0.088	0.170
BMI (kg/m^2^)	32.5 ± 7.4	32.9 ± 5.7	32.6 ± 5.8	35.5 ± 6.6	0.722	0.917	**0.002**	0.809	**0.002**	**0.005**
Neck circumference (cm)	41.2 ± 4.3	42.6 ± 3.7	42.3 ± 3.3	44.7 ± 4	0.058	0.236	**<0.001**	0.682	**0.001**	**0.003**
Waist circumference (cm)	112.4 ± 18.2	114.5 ± 13.7	117.2 ± 15	121.1 ± 15.1	0.477	0.226	**0.002**	0.399	**0.007**	0.221
Hip circumference (cm)	116.7 ± 16.8	115.4 ± 10.6	117.5 ± 13.4	121.2 ± 14.8	0.596	0.821	0.094	0.402	**0.008**	0.227
WHR	0.93 ± 0.06	0.98 ± 0.06	0.99 ± 0.07	1 ± 0.06	0.061	**0.020**	**0.001**	0.740	0.040	0.796
Smoking (%**)**	47.2	42.7	40.1	47.6	0.566	0.453	0.410	0.695	0.218	0.367

BMI: body mass index; WHR: waist to hip ratio.

**Table 2:** Comparison of sleep characteristics between controls and obstructive sleep apnea syndrome (OSAS) patients.

	Controls	Mild OSAS	Moderate OSAS	Severe OSAS	Controls versus mild OSAS	Controls versus moderate OSAS	Controls versus severe OSAS	Mild versus moderate OSAS	Mild versus severe OSAS	Moderate versus severe OSAS
TST (min)	294.7 ± 44.6	307.4 ± 43.8	302.9 ± 51.6	282.6 ± 73.6	0.224	0.505	0.097	0.699	**0.009**	**0.047**
N1 (%)	13.5 (10.8–20.6)	14.1 (7.9–25.2)	22.1 (15.2–32.2)	23.4 (13.2–40.6)	0.467	0.234	0.528	0.910	0.721	0.549
N2 (%)	55.1 ± 14.2	52.3 ± 17.5	52.1 ± 8.4	53.2 ± 12.2	0.471	0.323	0.466	0.929	0.787	0.577
N3 (%)	9.1 (2–17.1)	6.1 (0–12)	9.8 (3–16.9)	7.2 (3.2–17.8)	0.065	0.771	0.602	**0.047**	**0.005**	0.863
REM (%)	11 (7.3–17.9)	10 (6–23.5)	13 (9.3–17.2)	9.1 (4.7–14.7)	0.494	0.951	0.386	0.691	0.307	0.282
AHI (events/h)	2 (1–3.5)	10 (6–13)	21.3 (18–27)	61.4 (45–75.7)	**<0.001**	**<0.001**	**<0.001**	**<0.001**	**<0.001**	**<0.001**
ODI (events/h)	25.3 (14–46.7)	34.8 (10.8–50.7)	23.8 (12–60.2)	34.1 (12.7–68.7)	0.399	0.323	0.546	0.813	0.451	0.508
Aver. SpO_2_ (%)	92 ± 3.7	91.5 ± 4.1	92.2 ± 2.9	92.3 ± 2.7	0.547	0.863	0.713	0.444	0.200	0.862
Min. SpO_2_ (%)	76.7 ± 11	74.2 ± 15	80 ± 11	79.9 ± 10.2	0.419	0.242	0.123	0.091	**0.039**	0.976
Arousal index	18.1 (13.9–22.5)	22 (11.7–32.3)	27.9 (21.2–45.2)	23.1 (11.9–30.8)	0.166	0.310	0.388	0.484	**0.013**	0.055
Sleep efficiency (%)	78.5 ± 10.1	80 ± 9.4	80 ± 10.9	72.8 ± 15.8	0.514	0.572	**0.019**	0.998	**0.002**	**0.009**
ESS score	7.7 ± 5.6	8.9 ± 4.7	8.5 ± 5.2	10.8 ± 5.3	0.218	0.490	**0.001**	0.757	**0.018**	**0.034**

AHI: apnea-hypopnea index; Aver. SpO_2_: average oxyhaemoglobin saturation; ESS: Epworth sleepiness scale; Min. SpO_2_: minimum oxyhaemoglobin saturation; N1: sleep stage 1; N2: sleep stage 2; N3: sleep stage 3; ODI: oxygen desaturation index; REM: rapid eye movement; TST: total sleep time.

**Table 3:** Comparison of the laboratory measurements between controls and obstructive sleep apnea syndrome (OSAS) patients.

	Controls	Mild OSAS	Moderate OSAS	Severe OSAS	Controls versus mild OSAS	Controls versus moderate OSAS	Controls versus severe OSAS	Mild versus moderate OSAS	Mild versus severe OSAS	Moderate versus severe OSAS
FEV_1_ (% predicted)	99.3 ± 16.4	100.2 ± 18.3	100.4 ± 18.1	92.5 ± 21.9	0.787	0.786	0.051	0.964	**0.028**	0.086
FVC (% predicted)	94.4 ± 15.3	94.5 ± 15.5	94.7 ± 16.3	86.7 ± 20.8	0.963	0.929	**0.022**	0.958	**0.017**	0.067
FEV_1_/FVC (%)	84.9 ± 8.6	83.9 ± 9.1	86.1 ± 12.3	83.1 ± 12	0.553	0.616	0.341	0.356	0.662	0.256
pO_2_ (mmHg)	82.2 ± 14.2	82.7 ± 11.9	82.5 ± 11.6	76.4 ± 11	0.830	0.902	**0.008**	0.946	**0.001**	**0.008**
pCO_2_ (mmHg)	40.1 ± 3.4	40.3 ± 3.6	40.1 ± 3.7	41.7 ± 4	0.848	0.923	**0.020**	0.800	**0.033**	**0.045**
SBP (mmHg)	119.6 ± 14.3	125.9 ± 15.9	129.4 ± 15.5	134.8 ± 16.6	**0.008**	**<0.001**	**<0.001**	0.183	**<0.001**	**0.032**
DBP (mmHg)	77.7 ± 9.9	80.5 ± 8.7	80.1 ± 7.4	84 ± 9.5	0.052	0.105	**<0.001**	0.776	**0.004**	**0.002**
Cholesterol (mg/dL)	214.2 ± 45.9	210.7 ± 37.8	222.8 ± 47	229.1 ± 39.9	0.591	0.288	**0.010**	0.085	**<0.001**	0.317
Triglycerides (mg/dL)	130 (89–165)	162 (110.5–230)	144 (74–180.5)	120 (94–195)	**0.049**	0.712	0.467	0.144	0.148	0.663
LDL-C (mg/dL)	131.3 ± 35.2	131.6 ± 30.8	141.9 ± 39.3	129.9 ± 35.8	0.964	0.269	0.853	0.269	0.814	0.150
HDL-C (mg/dL)	50 ± 12.4	49.5 ± 14	50.9 ± 12.1	43.8 ± 10.4	0.830	0.649	**<0.001**	0.525	**<0.001**	**<0.001**
Glucose (mg/dL)	101.2 ± 21.5	103.8 ± 22.3	98.3 ± 20.2	107.1 ± 21.8	0.671	0.395	0.174	0.234	0.361	**0.047**
SCORE	0 (0–1)	1 (0–2)	1 (0–3)	1.5 (1–3)	**0.001**	**<0.001**	**<0.001**	**0.049**	**<0.001**	**0.023**
FRS	3 (1–5)	4 (1.5–7)	10 (2.5–13.5)	12 (5–15)	**0.004**	**<0.001**	**<0.001**	**0.030**	**<0.001**	**0.008**

DBP: diastolic blood pressure; FEV_1_: forced expiratory volume in 1st sec; FRS: Framingham Risk Score; FVC: forced vital capacity; HDL-C: high-density lipoprotein cholesterol; LDL-C: low-density lipoprotein cholesterol; pCO_2_: partial carbon dioxide pressure; pO_2_: partial oxygen pressure; SCORE: Systematic Coronary Risk Evaluation; SBP: systolic blood pressure.

**Table 4:** Association between Systematic Coronary Risk Evaluation (SCORE), Framingham Risk Score (FRS), and anthropometric and sleep parameters.

	SCORE		FRS	
	*r*	*p*	*r*	*p*
Age	0.652	**<0.001**	0.233	**<0.001**
BMI	−0.043	0.454	0.133	**0.019**
Neck circumference	−0.012	0.870	0.072	0.336
Waist circumference	−0.035	0.638	−0.029	0.701
Hip circumference	−0.061	0.417	−0.103	0.166
WHR	0.047	0.525	0.131	0.077
TST	−0.054	0.509	−0.188	**0.021**
N1	−0.007	0.927	0.035	0.668
N2	−0.007	0.933	0.028	0.732
N3	0.070	0.394	0.033	0.684
REM	−0.031	0.706	−0.122	0.135
AHI	0.251	**<0.001**	0.291	**<0.001**
ODI	0.049	0.661	−0.129	0.245
Aver. SpO_2_	0.131	0.107	0.132	0.104
Min. SpO_2_	0.127	0.119	0.143	0.102
Arousal index	−0.072	0.380	−0.127	0.122
Sleep efficiency	−0.073	0.372	−0.224	**0.006**
ESS score	0.103	0.156	0.108	0.135

AHI: apnoea hypopnea index; Aver. SpO_2_: average oxyhaemoglobin saturation; BMI: body mass index; ESS: Epworth sleepiness scale; Min. SpO_2_: minimum oxyhaemoglobin saturation; N1: sleep stage 1; N2: sleep stage 2; N3: sleep stage 3; ODI: oxygen desaturation index; REM: rapid eye movement; TST: total sleep time; WHR: waist to hip ratio.

## References

[1] (1999). “Sleep-related breathing disorders in adults: recommendations for syndrome definition and measurement techniques in clinical research: the report of an American Academy of Sleep Medicine Task Force. *Sleep*.

[2] Peppard P. E., Young T., Barnet J. H., Palta M., Hagen E. W., Hla K. M. (2013). Increased prevalence of sleep-disordered breathing in adults. *American Journal of Epidemiology*.

[3] Nena E., Steiropoulos P., Constantinidis T. C., Perantoni E., Tsara V. (2010). Work productivity in obstructive sleep apnea patients. *Journal of Occupational and Environmental Medicine*.

[4] He J., Kryger M. H., Zorick F. J., Conway W., Roth T. (1988). Mortality and apnea index in obstructive sleep apnea: experience in 385 male patients. *Chest*.

[5] Leung R. S., Bradley T. D. (2001). Sleep apnea and cardiovascular disease. *American Journal of Respiratory and Critical Care Medicine*.

[6] Pafili K., Steiropoulos P., Papanas N. (2015). The relationship between obstructive sleep apnoea and coronary heart disease. *Current Opinion in Cardiology*.

[7] Floras J. S. (2014). Sleep apnea and cardiovascular risk. *Journal of Cardiology*.

[8] Sanchez-de-la-Torre M., Campos-Rodriguez F., Barbe F. (2013). Obstructive sleep apnoea and cardiovascular disease. *The Lancet Respiratory Medicine*.

[9] (2013). “World Medical Association Declaration of Helsinki: ethical principles for medical research involving human subjects. *JAMA*.

[10] Marathe P. H., Gao H. X., Close K. L. (2017). American Diabetes Association Standards of Medical Care in Diabetes 2017. *Journal of Diabetes*.

[11] Tsara V., Serasli E., Amfilochiou A., Constantinidis T., Christaki P. (2004). Greek version of the Epworth Sleepiness Scale. *Sleep and Breathing*.

[12] Archontogeorgis K., Papanas N., Nena E. (2017). Insulin sensitivity and insulin resistance in non-diabetic middle-aged patients with obstructive sleep apnoea syndrome. *Open Cardiovascular Medicine Journal*.

[13] Iber C., American Academy of Sleep Medicine (2007). *The AASM Manual for the Scoring of Sleep and Associated Events: Rules, Terminology and Technical Specifications*.

[14] Conroy R. M., Pyorala K., Fitzgerald A. P. (2003). Estimation of ten-year risk of fatal cardiovascular disease in Europe: the SCORE project. *European Heart Journal*.

[15] D’Agostino R. B., Vasan R. S., Pencina M. J. (2008). General cardiovascular risk profile for use in primary care: the Framingham Heart Study. *Circulation*.

[16] Kiely J. L., McNicholas W. T. (2000). Cardiovascular risk factors in patients with obstructive sleep apnoea syndrome. *European Respiratory Journal*.

[17] Cintra F., Bittencourt L. R., Santos-Silva R. (2012). The association between the Framingham risk score and sleep: a Sao Paulo epidemiological sleep study. *Sleep Medicine*.

[18] Wang X. F., Li Q. Y., Wan H. Y., Li M. (2007). The influencing factors of Framingham cardiovascular risk score in patients with obstructive sleep apnea-hypopnea syndrome. *Zhonghua Yi Xue Za Zhi*.

[19] Loredo J. S., Nelesen R., Ancoli-Israel S., Dimsdale J. E. (2004). Sleep quality and blood pressure dipping in normal adults. *Sleep*.

[20] Mansoor G. A. (2002). Sleep actigraphy in hypertensive patients with the ‘non-dipper’ blood pressure profile. *Journal of Human Hypertension*.

[21] Simon R., Chirakalwasan N., Teerapraipruk B. (2012). Severity of obstructive sleep apnea in patients with and without cardiovascular-related diseases. *Respiratory Care*.

[22] Ross A. J., Yang H., Larson R. A., Carter J. R. (2014). Sleep efficiency and nocturnal hemodynamic dipping in young, normotensive adults. *American Journal of Physiology-Regulatory, Integrative and Comparative Physiology*.

[23] Bruno R. M., Palagini L., Gemignani A. (2013). Poor sleep quality and resistant hypertension. *Sleep Medicine*.

[24] Trinder J., Kleiman J., Carrington M. (2001). Autonomic activity during human sleep as a function of time and sleep stage. *Journal of Sleep Research*.

[25] Fung M. M., Peters K., Redline S. (2011). Decreased slow wave sleep increases risk of developing hypertension in elderly men. *Hypertension*.

[26] Le Bon O., Staner L., Hoffmann G. (2001). The first-night effect may last more than one night. *Journal of Psychiatric Research*.

[27] Archontogeorgis K., Nena E., Papanas N. (2015). Serum levels of vascular endothelial growth factor and insulin-like growth factor binding protein-3 in obstructive sleep apnea patients: effect of continuous positive airway pressure treatment. *Open Cardiovascular Medicine Journal*.

[28] Tzoulaki I., Seretis A., Ntzani E. E., Ioannidis J. P. (2014). Mapping the expanded often inappropriate use of the Framingham Risk Score in the medical literature. *Journal of Clinical Epidemiology*.

